# Continuation electroconvulsive therapy combined with pharmacotherapy for depression relapse prevention: A systematic review and meta-analysis

**DOI:** 10.1017/S0033291725101608

**Published:** 2025-08-28

**Authors:** Ana Jelovac, Richard Braithwaite, Charles H. Kellner, Declan M. McLoughlin

**Affiliations:** 1 Department of Psychiatry, School of Medicine, Trinity College Dublin, St Patrick’s University Hospital, Dublin, Ireland; 2 Sussex Partnership NHS Foundation Trust, Meadowfield Hospital, Worthing, UK; 3 Department of Psychiatry and Behavioral Sciences, Medical University of South Carolina, Charleston, SC, USA; 4 Trinity College Institute of Neuroscience, Trinity College Dublin, Dublin, Ireland

**Keywords:** bipolar disorder, continuation ECT, ECT, electroconvulsive therapy, maintenance ECT, major depressive disorder, relapse

## Abstract

Relapse following electroconvulsive therapy (ECT) remains a significant clinical challenge despite continuation of pharmacotherapy. We performed a systematic review and meta-analysis (PROSPERO CRD420251000113) of the efficacy and acceptability of continuation ECT (cECT) combined with pharmacotherapy compared to pharmacotherapy alone for relapse prevention following an acute course of ECT for depression. We searched PubMed, Embase, Web of Science, and CENTRAL databases for randomized controlled trials enrolling adults diagnosed with a unipolar or bipolar major depressive episode, who met remission or response criteria after an acute course of ECT and who were subsequently randomized to cECT with pharmacotherapy versus pharmacotherapy alone. The efficacy outcome was the cumulative relapse rate at 6-month follow-up. Data were synthesized using random-effects meta-analyses with effect sizes expressed as relative risks (RRs) with 95% confidence intervals (CIs). Four trials (*n* = 254) met the inclusion criteria. cECT combined with pharmacotherapy significantly reduced relapse compared to pharmacotherapy alone (RR = 0.57, 95% CI = 0.37–0.88; *I*^2^ = 0%; number needed to treat = 7). Sensitivity analyses consistently supported the superiority of cECT under all examined dropout scenarios and analytic approaches. Acceptability, measured by all-cause dropout, was similar between the groups (RR = 1.12; 95% CI = 0.48–2.62; *I*^2^ = 0%). cECT combined with pharmacotherapy significantly reduces the RR of relapse by 43% compared to pharmacotherapy alone without compromising acceptability. These findings reinforce the role of cECT as a valuable relapse prevention strategy following successful acute ECT and highlight the need for larger, multicenter trials to further optimize post-ECT prophylaxis.

## Introduction

Electroconvulsive therapy (ECT) is the most acutely effective treatment for depression, demonstrating significantly greater efficacy than anesthesia-only sham ECT (Meechan et al., 2022), oral antidepressants (UK ECT Review Group, 2003), ketamine (Rhee et al., 2025), and other neuromodulation therapies (Mutz et al., 2019). However, despite its unmatched acute efficacy, maintaining clinical remission following a successful acute course remains a key clinical challenge (Sackeim, 2017). Approximately half of all patients receiving continuation pharmacotherapy after successful ECT relapse within 1 year, with the majority of these relapses occurring within the initial 6 months (Jelovac et al., 2013). To address this high-risk period, more intensive treatment strategies, such as lithium augmentation (Lambrichts et al., 2021; Sackeim et al., 2001) and continuation ECT (cECT) (Brakemeier et al., 2014; Kellner et al., 2016; Kellner et al., 2006; Martínez-Amorós et al., 2021; Navarro et al., 2008; Nordenskjöld et al., 2013) have been investigated. cECT involves the ongoing administration of ECT sessions at gradually reduced frequency over weeks to months after the acute treatment phase. This gradual tapering of the frequency of ECT sessions prevents abrupt discontinuation of a previously effective treatment, with the intention of reducing the risk of early relapse. cECT administered for longer than 6 months following remission has been termed maintenance ECT and is designed to prevent recurrence of new episodes of depressive illness.

Previous systematic reviews on cECT versus pharmacotherapy for relapse prevention have yielded mixed and inconclusive results. A 2023 systematic review without meta-analysis identified 20 studies, mostly observational (Rowland et al., 2023). Prior meta-analyses (Dar et al., 2023; Elias et al., 2018; National Institute for Health and Care Excellence, 2022) of randomized trials either did not include all currently available trials or permitted inclusion of trials where cECT was used in a manner that would not reflect contemporary clinical practice (e.g. underdosed or used as monotherapy). This may have resulted in an underestimation of the efficacy of the widespread current practice of offering optimally dosed cECT in combination with pharmacotherapy.

The present systematic review and meta-analysis aims to provide an up-to-date evaluation of the efficacy and acceptability of cECT, as typically prescribed in real-world clinical practice (i.e. combined with pharmacotherapy), compared with pharmacotherapy alone, in preventing relapse following a successful acute course of ECT for depression.

## Methods

### Data sources

This systematic review, preregistered at PROSPERO (CRD420251000113), adhered to PRISMA 2020 guidelines (Page et al., 2021). We searched electronic databases and clinical trial registries (PubMed, Embase, Web of Science, and CENTRAL) on March 11, 2025, without date or language restrictions, using search terms provided in Supplementary Table S1. We also manually searched reference lists of eligible studies and prior systematic reviews. Search results were imported into and managed using Covidence.

Two reviewers (A.J. and R.B.) independently screened study abstracts. Abstracts deemed clearly ineligible according to both reviewers were excluded. For studies retained for full-text screening, the same two reviewers independently assessed eligibility. Discrepancies were resolved by discussion and consensus.

### Data extraction

Two reviewers (A.J. and R.B.) independently extracted data from published reports using a structured data extraction form. Extracted trial characteristics included first author, publication year, country, blinding, diagnostic criteria, entry criteria, outcome measures, intervention characteristics (ECT parameters and pharmacotherapy regimen), response/remission status at cECT baseline, definition of relapse, sample characteristics (age, sex, ethnicity, prevalence of bipolar disorder, and prevalence of psychotic features), sample size, number of relapses, and all-cause dropout rates. Discrepancies in data extraction were resolved by discussion and consensus. For unclear or incomplete data in the published reports, the authors of the original studies were contacted up to two times.

### Population

Included studies were randomized controlled trials enrolling adults (age ≥ 18 years) diagnosed with a unipolar or bipolar major depressive episode based on standardized diagnostic criteria (e.g. Feighner criteria, Research Diagnostic Criteria, Diagnostic and Statistical Manual of Mental Disorders [DSM], Third Edition through DSM, Fifth Edition, Text Revision, International Classification of Diseases, 9th Revision or 10th Revision). Eligible trials required participants to have achieved study-defined remission or response following acute ECT and to have been randomized thereafter to either cECT combined with pharmacotherapy or pharmacotherapy alone. Trials enrolling patients whose depressive symptoms were secondary to major medical conditions (e.g. cancer) or neurodegenerative disorders (e.g. Parkinson’s disease) were excluded, as were studies enrolling patients with schizophrenia or schizoaffective disorder.

### Interventions

Trials were eligible if they had a follow-up duration of at least 6 months comparing cECT combined with pharmacotherapy versus pharmacotherapy alone for relapse prevention after remission or response to acute ECT. Eligible studies administered cECT using any of the three electrode placements with the strongest evidence base in the acute phase: bitemporal (administered at any dose relative to seizure threshold), high-dose right unilateral (operationalized in the present meta-analysis as dose ≥5 times seizure threshold), and bifrontal (any dose relative to seizure threshold). Due to the dose-dependent acute efficacy of right unilateral ECT (Sackeim et al., 1987; Sackeim et al., 1993; Sackeim et al., 2000), with lower electrical stimulus doses relative to seizure threshold being less effective or ineffective, we assumed that the same applies to cECT. Thus, only trials utilizing high-dose right unilateral ECT relative to the individually titrated seizure threshold were included.

### Controls

Trials were included where both cECT and control groups received any active continuation pharmacotherapy strategy involving antidepressants and/or augmenting agents, including treatment-as-usual pharmacotherapy, individualized pharmacotherapy, and/or protocolized regimens.

### Outcomes

The primary efficacy outcome was cumulative relapse rate at 6 months following acute ECT, defined as the proportion of patients who had experienced a return of depressive symptoms up to and including the 6-month follow-up. A previous meta-analysis indicated that the highest risk of relapse following ECT occurs within the first 6 months (Jelovac et al., 2013), prompting the selection of this timeframe as our efficacy endpoint. We retained the original trial definitions of relapse, provided these used validated depression rating scales. Studies defining relapse solely based on clinical impression or psychiatric rehospitalization were excluded, as such criteria may underestimate true relapse rates.

The secondary outcome was acceptability, defined as the proportion of patients who withdrew from the study early or were noncompliant with randomized treatment for any reason. Tolerability in terms of cognitive side effects was not examined, as a prior 2022 meta-analysis reported no evidence of detrimental effects of cECT on cognitive outcomes (Yoldi-Negrete et al., 2022).

### Risk of bias assessment

Risk of bias was assessed by two independent reviewers (R.B. and D.M.M.) using the revised Cochrane Risk of Bias (RoB 2) tool (Sterne et al., 2019), evaluating bias related to the randomization process, deviations from intended interventions, missing outcome data, outcome measurement, and selective reporting of results. Discrepancies were resolved by discussion with a third reviewer (A.J.) and consensus.

### Statistical analyses

All outcomes were synthesized using random-effects meta-analyses with restricted maximum likelihood estimation, regardless of the magnitude of between-study heterogeneity as quantified by the *I*^2^ statistic (Higgins et al., 2003), owing to anticipated clinical variation among included studies in terms of patient populations, cECT schedules, and pharmacotherapy regimens. Both the primary efficacy endpoint (cumulative relapse rate at 6 months) and acceptability (all-cause dropout in the intention-to-treat (ITT) sample of all randomized participants during the 6-month continuation phase) were analyzed using this approach. Effect sizes were expressed as relative risks (RRs) with corresponding 95% confidence intervals (CIs). Prediction intervals were also calculated to estimate the range of effects expected in future studies (IntHout et al., 2016).

The primary efficacy analysis followed a modified ITT (mITT) approach, including all participants who initiated the assigned treatment by receiving at least one cECT session and/or medication dose, with missing data handled according to the last observation carried forward (LOCF) approach. Sensitivity analyses included: an mITT approach with worst-case imputation in which all patients who initiated the treatment and subsequently withdrew from the study were assumed to have relapsed; an ITT analysis with all randomized participants, regardless of treatment adherence, using LOCF; and a worst-case ITT analysis in which all post-randomization dropouts, including those who did not initiate the treatment, were assumed to have relapsed, providing a conservative estimate of treatment effect.

In additional sensitivity analyses, we applied the Hartung–Knapp–Sidik–Jonkman (HKSJ) adjustment to all efficacy models, a method commonly recommended in meta-analyses with a small number of studies (IntHout et al., 2014). While the HKSJ adjustment reduces the likelihood of type I error in small-sample meta-analyses, it can paradoxically produce narrower CIs when heterogeneity is very low, as observed here (Wiksten et al., 2016). As such, HKSJ models are reported as sensitivity analyses but not used for primary inference.

Due to the low number of included studies, subgroup analyses and meta-regressions were not conducted, and assessment of small-study effects (e.g. funnel plot asymmetry potentially indicating publication bias) was not feasible. Analyses were conducted using the *meta* (Balduzzi et al., 2019) and *robvis* (McGuinness & Higgins, 2021) packages in R version 4.4.3.

## Results

### Study selection

**Figure 1.**
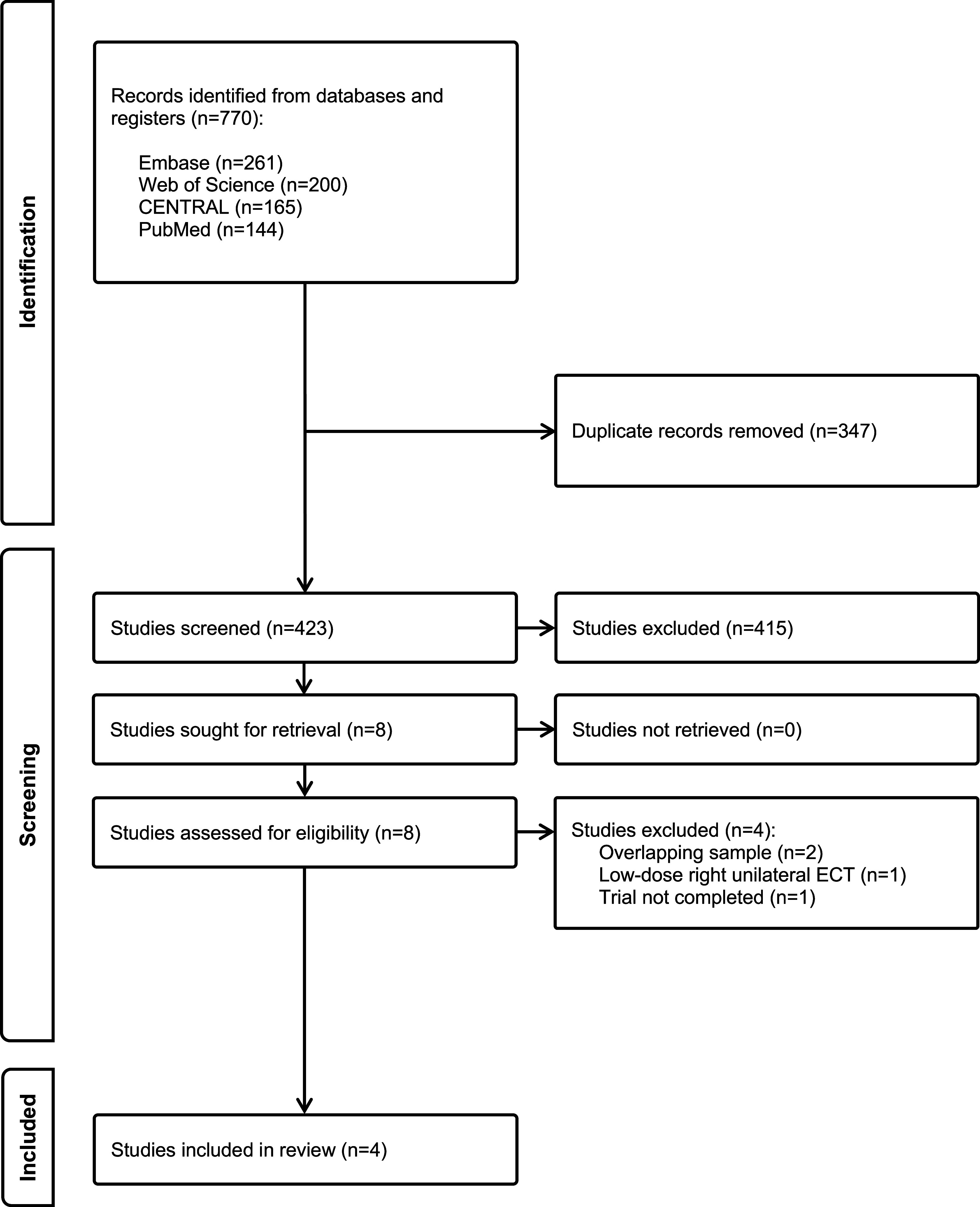
PRISMA 2020 flow diagram.

The systematic search identified 770 studies. After removal of database duplicates, 423 records underwent title and abstract screening. Of the eight studies retained for full-text screening, four trials met eligibility criteria (Kellner et al., 2016; Martínez-Amorós et al., 2021; Navarro et al., 2008; Nordenskjöld et al., 2013). Four records were excluded following full-text review: two had overlapping samples with included studies (Brus et al., 2024; Serra et al., 2006), one utilized low-dose right unilateral ECT (Brakemeier et al., 2014), and one planned study (trial registration: UMIN000006101) was never completed, according to the investigator. The study selection process is detailed in Figure 1.

### Study characteristics

Four trials with 254 randomized participants (127 per treatment arm) met eligibility criteria and were included in the meta-analysis (Table 1). Three trials were conducted in Europe (Martínez-Amorós et al., 2021; Navarro et al., 2008; Nordenskjöld et al., 2013), and one in North America (Kellner et al., 2016). All trials enrolled patients diagnosed with a DSM-IV major depressive episode who had achieved remission in three trials (Kellner et al., 2016; Martínez-Amorós et al., 2021; Navarro et al., 2008), or response in one trial (Nordenskjöld et al., 2013), following an acute course of ECT. Total sample sizes per study ranged from 33 to 128 randomized participants, though not all initiated the assigned treatment. Mean participant age was ~70 years in all but one study (Nordenskjöld et al., 2013). Two trials exclusively enrolled older adults (Kellner et al., 2016; Navarro et al., 2008). Participants in all trials were predominantly female; only one trial (Kellner et al., 2016) reported ethnic composition, which was predominantly White (95%). Three trials enrolled only patients with unipolar depression (Martínez-Amorós et al., 2021; Navarro et al., 2008; Nordenskjöld et al., 2013). Continuation pharmacotherapies consisted of individualized regimens in two trials (Martínez-Amorós et al., 2021; Nordenskjöld et al., 2013), nortriptyline or nortriptyline–risperidone combination in one trial (Navarro et al., 2008), and venlafaxine–lithium combination in another trial (Kellner et al., 2016). Two trials used brief pulse bitemporal cECT (Martínez-Amorós et al., 2021; Navarro et al., 2008), while another two trials used ultrabrief pulse right unilateral cECT at six times the seizure threshold (Kellner et al., 2016; Nordenskjöld et al., 2013). Follow-up durations ranged from 6 months to 2 years. Treatment characteristics and/or outcome data were not extractable from three published reports (Martínez-Amorós et al., 2021; Navarro et al., 2008; Nordenskjöld et al., 2013) but were provided by trial investigators via personal communication.

**Table 1.** Characteristics of eligible studies

Study	Country	Blinding	Acute phase, continuation phase, and relapse criteria	Acute ECT parameters	Continuation treatments	Sample size	Sample characteristics
Navarro et al. (2008)	Spain	Raters	Acute-phase entry criteria: age ≥ 60 years, DSM-IV unipolar MDE with psychotic features, HAM-D_17_ ≥ 21 Continuation phase entry criteria: remission (HAM-D_17_ ≤ 7 and resolution of psychotic features on three consecutive ratings) Relapse criteria: meeting DSM-IV criteria for MDE and HAM-D_17_ ≥ 16 on two consecutive ratings	Electrode placement: bifrontotemporal Dose: titrated to produce adequate seizure duration Pulse width: 1.0 ms Anesthesia: thiopental, succinylcholine, and atropine	cECT (same parameters as acute ECT; offered weekly for the first month, every 2 weeks for the following month, then monthly) combined with pharmacotherapy (nortriptyline [target blood level 80–120 ng/ml])	16 randomized and initiated	Age: mean 70.4 (SD 3.2) Female: 62.5% White: not reported Bipolar: 0% Psychotic: 100%
					Pharmacotherapy (nortriptyline [target blood level 80–120 ng/ml] and risperidone [up to 2 mg daily for 6 weeks, followed by a 4-week taper])	17 randomized and initiated	Age: mean 70.7 (SD 3.4) Female: 64.7% White: not reported Bipolar: 0% Psychotic: 100%
Nordenskjöld et al. (2013)	Sweden	No blinding	Acute-phase entry criteria: DSM-IV (MINI) unipolar or bipolar MDE Continuation phase entry criteria: response (CGI-I score 1 or 2 and MADRS ≤15) Relapse criteria entry criteria: MADRS ≥20, psychiatric hospitalization, suicide, or suspected suicide	Electrode placement: mostly right unilateral (86% of patients), with switching to bifrontotemporal or bifrontal permitted Dose: mostly age- and sex-based dosing (mean charge 443 mC [SD = 239 mC]) Pulse width: ranging from 0.3 to 1.0 ms Anesthesia: propofol or thiopental, succinylcholine, and glycopyrrolate	cECT (mostly ultrabrief pulse [mean pulse width 0.36 ms] right unilateral at 6xST; offered weekly for 6 weeks, then every 2 weeks) combined with individualized pharmacotherapy	28 randomized; 26 initiated	Age: mean 52 (SD 17) Female: 57% White: not reported Bipolar: 21% Psychotic: 36%
					Individualized pharmacotherapy	28 randomized and initiated	Age: mean 62 (SD 13) Female: 43% White: not reported Bipolar: 14% Psychotic: 39%
Kellner et al. (2016)	United States	Raters	Acute-phase entry criteria: age ≥ 60 years, DSM-IV (SCID or MINI) unipolar MDE, HAM-D_24_ ≥ 21 Continuation phase entry criteria: remission (HAM-D_24_ ≤ 10 on two consecutive ratings and no increase in HAM-D_24_ score of >3 points on the second consecutive rating, or the score remained ≤6) Relapse criteria: HAM-D_24_ ≥ 21 on two consecutive ratings, psychiatric hospitalization, or suicidality	Electrode placement: right unilateral Pulse width: 0.25 or 0.3 ms Dose: 6xST Anesthesia: methohexital and succinylcholine; glycopyrrolate used only at the dose titration session	cECT (same parameters as acute ECT; initial fixed component of four cECT treatments in 1 month, followed by flexible treatment frequency in weeks 5–24 determined by application of a symptom-driven algorithm based on the patient’s HAM-D_24_ scores) combined with pharmacotherapy (venlafaxine [target dose 225 mg] and lithium [target blood level 0.4–0.6 mmol/L])	64 randomized; 61 initiated	Age: mean 70.8 (SD 7.2) Female: 60.7% White: 95.1% Bipolar: 0% Psychotic: 8.2%
					Pharmacotherapy (venlafaxine [target dose 225 mg] and lithium [target blood level 0.4–0.6 mmol/L])	64 randomized; 59 initiated	Age: mean 70.3 (SD 7.3) Female: 62.7% White: 94.9% Bipolar: 0% Psychotic: 20.3%
Martínez-Amorós et al. (2021)	Spain	Raters	Acute-phase entry criteria: DSM-IV-TR unipolar MDE Continuation phase entry criteria: remission (HAM-D_21_ ≤ 7 on two consecutive ratings) Relapse criteria: HAM-D_21_ score of 15–17 on two consecutive ratings or HAM-D_21_ ≥ 18 on a single rating	Electrode placement: bifrontotemporal Pulse width: 1.0 ms Dose: half-age dosing method Anesthesia: thiopental and succinylcholine	cECT (same parameters as acute ECT, offered weekly for 4 weeks, every 2 weeks for the following two months, then monthly) combined with individualized pharmacotherapy	19 randomized; 18 initiated	Age: median 69 (range 34–81) Female: 72.2% White: not reported Bipolar: 0% Psychotic: 44.5%
					Individualized pharmacotherapy	18 randomized and initiated	Age: median 67 (range 52–77) Female: 61.1% White: not reported Bipolar: 0% Psychotic: 72.3%

Abbreviations: cECT, ‘continuation electroconvulsive therapy’; CGI-I, ‘Clinical Global Impression–Improvement’; DSM-IV, ‘Diagnostic and Statistical Manual of Mental Disorders, Fourth Edition’; DSM-IV-TR, ‘Diagnostic and Statistical Manual of Mental Disorders, Fourth Edition, Text Revision’; ECT, ‘electroconvulsive therapy’; HAM-D_17_, ‘17-item Hamilton Depression Rating Scale’; HAM-D_21_, ‘21-item Hamilton Depression Rating Scale’; HAM-D_24_, ‘24-item Hamilton Depression Rating Scale’; MADRS, ‘Montgomery–Åsberg Depression Rating Scale’; mC, ‘millicoulomb’; MINI, ‘Mini-International Neuropsychiatric Interview’; MDE, ‘major depressive episode’; SCID, ‘Structured Clinical Interview for DSM-IV Disorders’; SD, ‘standard deviation’; ST, ‘seizure threshold’.

### Efficacy

**Figure 2.**
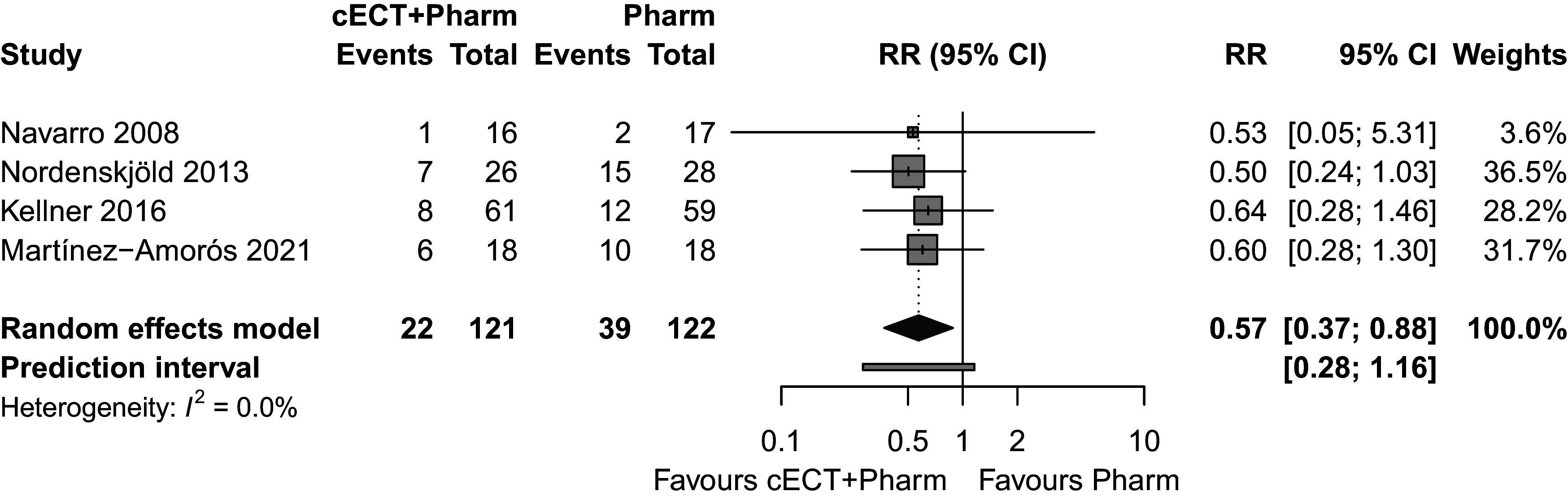
Efficacy of cECT and pharmacotherapy combination versus pharmacotherapy alone in 6-month relapse prevention.

In the primary mITT analysis of four eligible trials (Figure 2), which included all participants (*n* = 243) who received at least one cECT session and/or medication dose, cECT combined with pharmacotherapy significantly reduced the risk of relapse compared to pharmacotherapy alone (RR = 0.57; 95% CI = 0.37–0.88; prediction interval = 0.28–1.16; *I*^2^ = 0%). This corresponds to an RR reduction of 43% and a number needed to treat of 7 (95% CI = 5–24), indicating that for every seven patients treated with cECT plus pharmacotherapy, one additional relapse is prevented compared to pharmacotherapy alone. Results remained statistically significant with HKSJ adjustment (Supplementary Table S2).

Sensitivity analyses using mITT (*k* = 4; *n* = 243) and ITT (*k* = 4; *n* = 254) approaches with different methods of handling missing data (LOCF and worst-case scenarios), with and without HKSJ adjustment, yielded statistically significant effects favoring cECT, consistently observed across all analytic populations and statistical methods (Supplementary Table S2). No between-study heterogeneity was observed in any sensitivity analysis (*I*^2^ = 0%). Although statistically significant effects in favor of cECT were observed across all efficacy analyses, wider prediction intervals crossing 1 suggest the possibility that the observed benefit may not consistently achieve statistical significance across all future clinical settings.

### Acceptability

**Figure 3.**
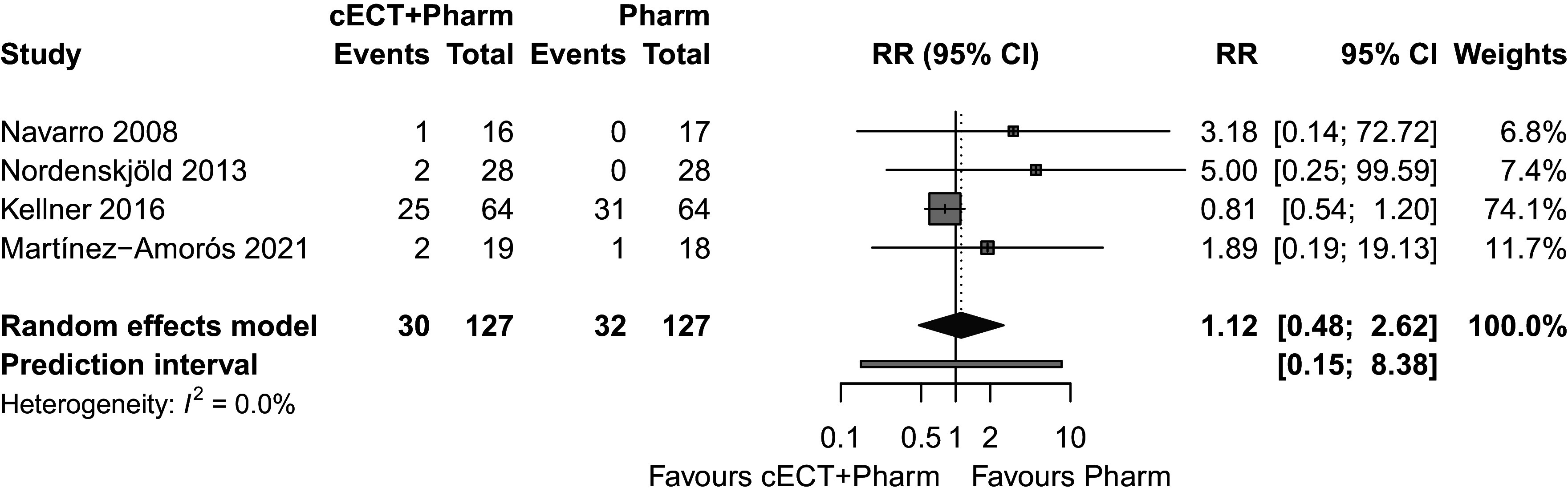
Acceptability of cECT and pharmacotherapy combination versus pharmacotherapy alone.

All-cause dropout rates did not significantly differ between groups (RR = 1.12; 95% CI = 0.48–2.62; prediction interval = 0.15–8.38; *I*^2^ = 0%), indicating that cECT combined with pharmacotherapy was no less acceptable than oral pharmacotherapy alone (Figure 3). However, the wide prediction interval indicates substantial uncertainty regarding real-world acceptability.

### Risk of bias

Risk of bias was assessed using the RoB 2 tool. Among the four included trials, two (Martínez-Amorós et al., 2021; Navarro et al., 2008) had some concerns due to a lack of information regarding allocation concealment. The remaining two trials (Kellner et al., 2016; Nordenskjöld et al., 2013) were rated as having a high risk of bias due to deviations from the intended interventions, missing data, and/or outcome measurement. Detailed risk of bias ratings for each study and domain are presented in Supplementary Figures S1 and S2.

## Discussion

This meta-analysis demonstrated that cECT, when administered in combination with continuation pharmacotherapy, significantly reduced the RR of 6-month relapse by 43% compared with pharmacotherapy alone following successful acute ECT for depression. This therapeutic benefit was achieved without compromising acceptability relative to oral pharmacotherapy prophylaxis alone. Importantly, effect sizes were consistent across all eligible trials despite marked variability in cECT parameters, pharmacotherapy regimens, and patient populations. No statistical heterogeneity was observed in any analysis, and all sensitivity analyses affirmed the robustness of the primary efficacy results against variations in analytic approaches and handling of missing data.

Although based on a modest sample size, our findings align with those from large-scale observational studies, including a 2024 Danish nationwide cohort study of 19,944 patients who received a course of ECT over 20 years for a range of psychiatric diagnoses (Jørgensen et al., 2024). In propensity score-matched analyses, cECT was associated with significantly reduced psychiatric rehospitalization (hazard ratio [HR] = 0.69; 95% CI = 0.56–0.84) and suicidal behavior (HR = 0.47; 95% CI = 0.27–0.82) within 6 months of acute ECT (Jørgensen et al., 2024). For patients with unipolar depression, the HR for rehospitalization was 0.66 (95% CI = 0.55–0.78) (A. Jørgensen, personal communication). Our findings, however, contrast with those of two prior meta-analyses (Dar et al., 2023; National Institute for Health and Care Excellence, 2022) that permitted the inclusion of trials in which cECT was delivered in ways inconsistent with contemporary clinical practice. The National Institute for Health and Care Excellence (2022) meta-analysis, which underpins its current clinical guidance on cECT, included only two trials (Brakemeier et al., 2014; Kellner et al., 2016), one of which used low-dose right unilateral ultrabrief pulse ECT (Brakemeier et al., 2014). Two other randomized trials available at the time were excluded: in one instance, because more than 20% of participants exhibited psychotic symptoms at acute ECT baseline (Nordenskjöld et al., 2013), and in the other due to the timing of randomization (Navarro et al., 2008). Such exclusions eliminated populations most likely to receive and benefit from cECT in typical clinical settings, thereby limiting the clinical relevance of findings.

In contrast, the present meta-analysis applied stringent, clinically grounded inclusion criteria, focusing exclusively on trials of cECT delivered at adequate stimulus doses and used as an adjunct to pharmacotherapy. Among the four trials included, three reported a mean participant age of ~70 years and substantial proportions of patients with psychotic depression. Both higher age and the presence of psychotic features are associated with enhanced therapeutic response (van Diermen et al., 2018) and lower relapse risk (Jelovac et al., 2013) following ECT. Consequently, the observed benefits of cECT documented in the present meta-analysis are most relevant to this prototypical ECT clinical population. Generalization to younger patients or those with chronic, treatment-resistant, or complex presentations, including comorbid anxiety disorders, personality disorders, or affective instability, may be limited.

The optimum frequency schedule for cECT has not yet been established. The studies included in the present systematic review used a variety of schedules over the 6-month treatment period. One trial stopped prescheduled treatments after the first month, adding subsequent ad hoc sessions in the event of reemerging symptomatology (Kellner et al., 2016). Two trials used a steadily decreasing frequency that was reduced to monthly after 8 weeks (Martínez-Amorós et al., 2021; Navarro et al., 2008), while one trial used a more cautious protocol that did not reduce cECT frequency to lower than fortnightly (Nordenskjöld et al., 2013). A survey of ECT clinics in the global north found that these latter approaches are the most common in routine practice, with 85% of clinics steadily reducing the frequency of scheduled treatments, although the typical speed of these reductions is unclear (Rohde et al., 2024). This gradual reduction in cECT frequency is a sensible clinical strategy given that the risk of relapse is greatest in the initial few months following cessation of acute ECT (Jelovac et al., 2013).

The prevalence of cECT usage following an acute course varies considerably between countries. An otherwise comprehensive review (Daskalakis et al., 2024) of ECT practice in low- and middle-income countries did not mention cECT, suggesting its use may be rare. In the United Kingdom and the Republic of Ireland, 1,720 acute and 80 cECT courses were administered for depression in the year 2022, indicating that fewer than 5% of acute ECT courses are followed by a continuation course (Royal College of Psychiatrists, n.d.). As of 2013, 11% of patients in the Swedish National Quality Register for ECT (Nordanskog et al., 2015) and 27% in a Finnish survey received cECT (Sumia et al., 2021). Finally, a survey of providers undertaken in 2022, predominantly covering North America, found that 97% of clinics offered cECT, and that a median of 50% of patients completing acute ECT courses received this form of continuation treatment (Rohde et al., 2024).

The present meta-analysis has several limitations. The small number of eligible trials (*k* = 4) precluded a formal assessment of funnel plot asymmetry, subgroup analyses, or meta-regression to evaluate the potential influence of ECT modality, dosing strategies, or patient characteristics. Nonetheless, the absence of statistical heterogeneity across all analyses indicates consistent treatment effects, suggesting minimal value from additional exploratory analyses. Second, an inherent limitation of all cECT trials conducted to date is the lack of participant blinding. Recruitment into long-term double-blind trials in this area would be challenging as it would require multiple sham ECT sessions involving repeated general anesthesia. Although three of the four included trials employed blinded outcome assessors, the findings should be interpreted in the context of the fundamentally distinct treatment experiences between the groups, with the cECT group receiving more intensive medical attention. Finally, although previous meta-analytic estimates of the 6-month relapse rate in schizophrenia and depression were remarkably similar – 36.9% (Aoki et al., 2024) versus 37.7% (Jelovac et al., 2013) – our findings should not be generalized to ECT patients with diagnoses beyond unipolar and bipolar depression (e.g. schizophrenia, schizoaffective disorder, or mania), as these populations were not represented in the trials included in the present meta-analysis.

## Conclusion

In conclusion, adequately dosed cECT combined with pharmacotherapy is an effective relapse prevention strategy following successful acute ECT for depression. Considering the limited evidence available from clinical trials, further definitive, multicenter randomized controlled trials are necessary to confirm these findings and to further optimize long-term relapse prevention strategies for patients responding to ECT.

## Supporting information

Jelovac et al. supplementary material Jelovac et al. supplementary material

## Supplementary material

The supplementary material for this article can be found at http://doi.org/10.1017/S0033291725101608.
